# Supplementary material for: Stacking Model‐Based Classifiers for Dealing With Multiple Sets of Noisy Labels
Source: Biom J. 2025 Mar 12;67(2):e70042. doi: 10.1002/bimj.70042 (PMC11898607; doi:10.1002/bimj.70042)
Supplement: Supplementary file 1 — Supporting Information [file BIMJ-67-e70042-s001.zip › density-based-ensemble-model-main/competitors_real_data/netlab-master/netlab-master/help/gtmfwd.htm]

Netlab Reference Manual gtmfwd


# gtmfwd

## Purpose

Forward propagation through GTM.

## Synopsis

```
mix = gtmfwd(net)
```

## Description

`mix = gtmfwd(net)` takes a GTM
structure `net`, and forward
propagates the latent data sample `net.X` through the GTM to generate
the structure
`mix` which represents the Gaussian mixture model in data space.

## See Also

`gtm`

---

**Pages:**
Index


---

Copyright (c) Ian T Nabney (1996-9)
